# Perceived Physical Education Teachers’ Controlling Behaviour and Students’ Physical Activity during Leisure Time—The Dark Side of the Trans-Contextual Model of Motivation

**DOI:** 10.3390/bs12090342

**Published:** 2022-09-16

**Authors:** Andres Viksi, Henri Tilga

**Affiliations:** Institute of Sport Sciences and Physiotherapy, Faculty of Medicine, University of Tartu, 51008 Tartu, Estonia

**Keywords:** perceived controlling behaviour, basic psychological need frustration, controlled motivation, autonomous motivation, physical education, physical activity

## Abstract

Background: Previous studies have shown that the controlling behaviour of physical education teachers might be detrimental to their students’ psychological experiences. The purpose of this work was to examine whether and to what extent the different dimensions of the perceived controlling teaching questionnaire predict students’ basic psychological needs, motivations, and physical activities during leisure-time. Methods: A total of 299 students (164 boys and 135 girls) from four Estonian general education schools and two vocational education institutions participated in the study. Students filled in the questionnaire of study variables. A variance-based structural model was used to test the research hypotheses. Results: The results revealed that different forms of controlling behaviours predicted psychological need frustration (β = 0.09–0.37; *p* < 0.01). Psychological need frustration predicted controlled motivation (β = 0.52; *p* < 0.01). Controlled motivation predicted subjective norms (β = 0.51; *p* < 0.01). Intention was predicted by attitudes (β = 0.30; *p* < 0.01), perceived behavioural control (β = 0.37; *p* < 0.01), and subjective norms (β = 0.15; *p* < 0. 01). Attitude was statistically significantly related to leisure-time physical activity (β = 0.09; *p* < 0.05). The model describes 10% of students’ physical activity in the context of leisure-time. Conclusion: The results of this study highlight that physical education teachers should avoid using controlling behaviours if the aim is to avoid frustrating their students’ psychological needs, which might have detrimental effect on students’ leisure-time physical activity via controlled forms of motivation.

## 1. Introduction

### 1.1. Theoretical Background

#### 1.1.1. Trans-Contextual Model of Motivation

One of the main goals of physical education is to share physical skills, knowledge, and competencies with students so that they can engage in activities that require physical activity in their leisure-time. Whereas in the past the goal of physical education was improving physical ability and the successful performance of activities, today, it is important to maintain health and ensure it through physical activity. It is complicated to predict whether a physical education teacher will shape a young person’s physical habits. The model we used in the present study to explain students’ physical activity is the trans-contextual model of motivation [1]. One of the aims of the model is to explain how motivation is transferred from one context (e.g., physical education) to another context (e.g., leisure-time) [2]. It is a multi-theoretical approach consisting of three socio-cognitive theories of motivation: the theory of self-determination [3], the theory of planned behaviour [4], and, finally, Vallerand’s hierarchical model, which combines the theory of self-determination and the theory of planned behaviour [5].

#### 1.1.2. Self-Determination Theory

The self-determination theory is a theory of human motivation and personality in a social context that distinguishes between different forms of motivation such as autonomous and controlled forms of motivation [1]. The three main components of self-determination are the three universal psychological needs, confirmed through decades of empirical work. There is a need for competence (i.e., a person feels that he or she is good at something and applies it), a need for autonomy (i.e., a person has choices and responsibilities), and a need for relatedness (i.e., a person feels that someone is connected with him or her) [1].

Teaching that is perceived as autonomy-supportive satisfies the students’ basic psychological needs for autonomy, competence, and relatedness [6]. This teaching style is related to the autonomous functioning of students. Autonomous means that students are supported in their choices, decision-making, and responsibilities [7]. Recent studies have also extensively found that perceived autonomy support could be categorized between three dimensions such as cognitive, organisational, and procedural [8,9,10,11]. Based on students’ perceptions, several intervention studies have demonstrated that physical education teachers can effectively learn how to become autonomy-supportive [12,13]. Studies have also found that autonomy-supportive teaching is beneficial not only to students’ outcomes but to teachers themselves, who benefit from adopting an autonomy-supportive style [14]. Controlling teaching, on the other hand, limits students’ basic psychological needs and leads to the frustration of basic psychological needs and an inability to adapt [15]. Recent studies have also found that perceived controlling behaviour is detrimental to autonomy-supportive behaviours [16], and perceived autonomy support and controlling behaviour might affect students’ outcomes via different pathways [17]. These findings highlight the need to understand the dark pathway in the motivational processes.

#### 1.1.3. The Theory of Planned Behaviour

The theory of planned behaviour is a further development of the earlier theory of reasoned action [4]. As with the theory of reasoned action, the individual’s intention to behave in some way is central. Intentions are expected to be related to motivational factors that influence behaviour. Intention is an indicator of how much a person is striving to perform. Intention is related to three indicators: attitude, subjective norms, and perceived behavioural control. Perceived behavioural control is also an indicator that distinguishes the theory of reasoned action and the theory of planned behaviour [4,18]. Attitude towards behaviour indicates whether an individual evaluates a certain behaviour positively or negatively. The subjective norm indicates whether an individual perceives social pressure to perform certain behaviours. Perceived behavioural control refers to an individual’s past experiences related to certain behaviours [4,18].

#### 1.1.4. Vallerand’s Hierarchical Model

Vallerand’s [5] hierarchical model connects the theory of self-determination with the theory of planned behaviour. Vallerand’s hierarchical model identifies the factors influencing motivation: situational, contextual, and global. Situational motivation shows what an individual experiences during a certain activity at a certain point in time. Contextual motivation indicates that an individual may be motivated in a particular area but may lack motivation in other areas. Global motivation indicates whether an individual behaves because they are motivated by internal motives, external motives, or no motivation at all [5].

#### 1.1.5. Controlling Behaviour of a Teacher Perceived by a Student in Physical Education

Each teacher and their teaching style is different. Some teachers support learner-centredness. The teacher sees the students in perspective, encourages them to make an effort, and is ready to give students choices so that the students can choose the most suitable topic or direction. Other teachers teach what they think is right and best and do not leave students any choice. They put pressure on students to think, behave, and feel the way they think is best [7].

Physical education teachers believe that the effects of teaching styles that support or control autonomy only affect students who achieve better results in terms of autonomous or controlled motivation, respectively. Contradictions with teachers’ perceptions were shown by an experimental study on students, in which students with low levels of motivation indicated that they would benefit from a teaching style that supported autonomy and would suffer from a controlling teaching style. Thus, if teachers want to promote the intrinsic motivation and success of their students, it would be good for them to adopt an attitude that supports autonomy, even if their students appear to be motivated or motivated in a controlled manner [19]. Previous studies have found that the experience of intrinsic motivation is crucial for adolescents’ daily physical activity [20]. On the other hand, perceived controlling behaviour might be detrimental to adolescents’ well-being [21,22].

#### 1.1.6. Physical Activity of Students in Physical Education

For children aged 5–17 years, 60 min of daily, moderate-to-vigorous physical activity can be considered the recommended level of physical activity [23]. However, a recent study found that only 2.5% of students met the 60 min MVPA per day recommendation for 7 measured days, whereas 14.6% of students did not meet the recommendation on any of the days [20]. Adolescents have physical education classes approximately 2 to 3 times a week, but this is not enough, and young people should be physically active during leisure-time [23]. More PE classes are needed to teach the importance of PA to students to increase their overall MVPA. Using a trans-contextual model of motivation, recent research has found that perceived controlling behaviours might be detrimental to adolescents’ physical activity via the motivations and components of the theory of planned behaviour [24,25]. On the other hand, the trans-contextual model of motivation-based research has also found that autonomy support might have a positive impact on adolescents’ leisure-time physical activities [26,27].

### 1.2. The Present Study

The main aim of this study was to examine whether and to what extent the different dimensions of perceived controlling behaviours predict the need frustrations, motivations, and physical activities of students in a leisure-time context. The survey was conducted in Estonian schools among students from 7-to-12-year-old classes. Gender differences in the study variables were further examined as one of the sub-objectives. Based on the main aim of the study, five sub-goals were set:

1. Examining how the different dimensions of the Externally and Internally Controlling Teaching Scale (EICT) questionnaire predict the frustration of students’ basic psychological needs.
2. Examining how the EICT questionnaire and the CCBS questionnaire predict the components of the trans-contextual model of motivation.
3. Examining how the different dimensions of the EICT and CCBS questionnaires predict student motivation in physical education through the frustration of students’ basic psychological needs in physical education.
4. Examining how the different dimensions of the EICT and CCBS questionnaires predict students’ physical activities in their leisure-time due to need frustration and the motivation of students’ basic psychological needs during physical education.
5. Examining if there are significant differences in the variables of the study between boys and girls.

## 2. Method

### 2.1. Participants and Study Design

The sample of the present research consisted of 299 basic school and secondary school students (164 boys and 135 girls) aged 13 to 18 years old (M = 16.10; SD = 1.86) from vocational schools and general education schools in Viljandi County, Estonia, who were invited to participate in the survey on a voluntary basis. Six schools in Viljandimaa gave their consent, which were Viljandi Vocational Training Centre, Olustvere School of Service and Rural Economics, Suure-Jaani School, Suure-Jaani Gymnasium, August Kitzberg Gymnasium, and Olustvere Basic School.

A researcher communicated with the physical education teachers in the schools about their agreement to take part in the survey. The school management was sent information about the survey, what the survey was about, and which students would be included in the survey and how. With the consent of the management, information was sent to the physical education teachers at the schools, which they passed on to the children and through the children to the parents. With the consent of the parents, a questionnaire was sent to the students, which they completed either in physical education classes or in their leisure-time. Students were introduced to the objectives of the survey before the survey was conducted, anonymity was emphasized, and it was said that it was possible to quit at any time.

The survey was conducted in the Google Forms environment, where the survey forms were completed. Respondents were not asked to identify themselves. It took about 15 min to fill in the survey. The survey consisted of the following various scales used as measuring instruments. The approval of the design and procedures of the present study was obtained from the Research Ethics Committee of the University of Tartu, Estonia (332/T-28).

### 2.2. Measures

#### 2.2.1. Perceived Internal and External Controlling Teaching

A 2-dimensional, 12-statement EICT questionnaire [19] was used to measure the internally and externally controlling teaching of physical education teachers perceived by the students. The questionnaire measures the internally and externally controlling teaching perceived by students. The questionnaire consists of two subscales (i.e., internally and externally controlling teaching), each with 6 statements. Each statement had to be rated on a 7-point scale from 1 (strongly disagree) to 7 (strongly agree). The statements used in the questionnaire were, for example: “My physical education teacher counts down aloud to make sure that I persist” (externally controlling) and “My physical education teacher pays less attention to me when I disappoint him/her” (internally controlling). Previous research has shown that the perceived scale of internally and externally controlling teaching is valid and reliable [28,29,30].

#### 2.2.2. Multidimensional Controlling Teaching Perceived by Students

A 12-item questionnaire (the Multidimensional Controlling Coach Behavior Scale (CCBS) [21]), adapted and validated in the context of physical education [22], was used to assess students’ perceived multidimensional controlling teaching. The questionnaire consists of 3 subscales (i.e., negative conditional regard, intimidation, controlling use of grades), each with 3 statements. Each statement had to be rated on a 7-point scale from 1 (strongly disagree) to 7 (strongly agree). The statements used in the questionnaire were, for example: “My physical education teacher is less friendly to me if I don’t make as much effort as he wants” (negative conditional regard), “My physical education teacher tries to motivate me by promising a good grade only” (controlling use of grades), and “My physical education teacher threatens to punish me if I don’t practice in class” (intimidation). Previous research has shown that the perceived scale of multidimensional controlling teaching is valid and reliable [21,22]. The results of two subscales (negative conditional regard and intimidation) were used in the present work, as they are the most relevant in physical education and showed associations with frustration in the study [22].

#### 2.2.3. Frustration of the Basic Psychological Needs

A 12-item questionnaire (Basic Psychological Need Satisfaction and Need Frustration scale [15]), adapted and validated in the context of physical education [31], was used to assess students’ basic psychological need frustration. The questionnaire measures students’ frustrations with autonomy, competence, and relatedness in physical education. The subscales of the questionnaire consist of 4 statements. Each statement had to be rated on a 7-point scale from 1 (strongly disagree) to 7 (strongly agree). The questions used in the work were, for example, “In physical education I felt compelled to do a lot of exercises that I would not have chosen to do” (i.e., autonomy), “In physical education I had serious doubts about whether I could do the exercises well” (i.e., competence), and “In physical education, I felt that my classmates who were important to me were indifferent to me and kept me away” (i.e., relatedness). Previous research has shown that the scale for assessing basic psychological need frustration is valid and reliable [32,33].

#### 2.2.4. Perceived Autonomous and Controlled Motivation of Students in Physical Education

An 8-item questionnaire (Perceived Locus of Causality Questionnaire [34]) was used to measure students’ perceived autonomous and controlled motivation in the context of physical education. The questionnaire measures students’ internal motivation, external motivation, identified regulation, and introjected regulation. In this study, the authors did not include statements on integrated regulation because statements on integrated regulation are more relevant to older students. There were 2 statements in each subscale of the questionnaire. The statements had to be rated on a 7-point scale from 1 (strongly disagree) to 7 (strongly agree). Examples used in the work were: “I take part in a physical education class because I have to do it, that’s the norm” (i.e., external motivation), “I take part in a physical education class because I would feel bad if I didn’t do it” (i.e., introjected regulation), “I attend physical education class because it is important for me to improve my skills in class” (i.e., identified regulation), and “I participate in physical education class because I like physical education” (i.e., intrinsic motivation). The averages of the internal motivation and identified regulatory subscale values were used to construct the characteristic autonomic motivation in the context of physical education, and the averages of the external motivation and introjected regulatory subscale values were used to generate the characteristic controlled motivation in the context of physical education. Previous studies show that the scale of perceived autonomous and controlled motivation in the context of physical education is valid and reliable [35], and the scale has been used previously in Estonia [25,26].

#### 2.2.5. Perceived Autonomous and Controlled Motivation of Students in Their Leisure Time

An earlier adapted version of Ryan and Connell’s [36] 8-item questionnaire was used to measure the students’ perceived autonomy and controlled motivation in a leisure-time context. The questionnaire measures students’ internal motivation, external motivation, identified regulation, and introjected regulation. In this study, the authors did not include statements on integrated regulation because statements on integrated regulation are more relevant to older students. There were 2 statements in each subscale of the questionnaire. The statements had to be rated on a 7-point scale from 1 (strongly disagree) to 7 (strongly agree). Examples used in the work were: “I am physically active in my leisure time because I value the benefits of physical activity” (i.e., intrinsic motivation), “I am physically active in my leisure time because people who know me would not be happy with me if I were not physically active” (i.e., introjected motivation), “I am physically active in my leisure time because it is important for me to be physically active” (i.e., identified motivation), and ”I am physically active in my leisure time because I feel the pressure of people who know me to be physically active” (i.e., external motivation). The averages of the internal motivation and identified regulatory subscale values were used to construct the characteristic autonomic motivation in the leisure context, and the averages of the external motivation and introjected regulation subscale values were used to generate the attribute-controlled motivation in the leisure context. Previous studies show that the scale of perceived autonomous and controlled motivation in the context of leisure is valid and reliable [37], and the scale has been used previously in Estonia [25,26].

#### 2.2.6. The Theory of Planned Behaviour

A 7-statement questionnaire was used to measure the features of the planned behaviour theory, for which a separate guide has been developed [38]. The questionnaire measures intention, attitude, perceived behavioural control, and subjective norms. The intention consists of 2 statements (e.g., “I intend to do sports and/or strenuous physical activity in the leisure-time in the next 5 weeks”), which are rated on a 7-point scale from 1 (strongly disagree) to 7 (strongly agree). Attitude consists of one statement (“For me, engaging in sports and/or strenuous physical activity in the leisure-time for the next 5 weeks is…”), which is rated on 3 different 7-point scales ranging from 1 (e.g., unpleasant) to 7 (e.g., pleasant); perceived behavioural control consisted of two items (e.g., “How much control do you have over yourself to do sports and/or strenuous exercise in your leisure-time over the next 5 weeks?”) on a 7-point scale from 1 (very low) to 7 (full). The subjective norm consists of two statements (e.g., “Most people who are important to me think that I should do sports and/or strenuous physical activity in my spare time in the next 5 weeks.”), which are evaluated on a 7-point scale from 1 (strongly disagree) to 7 (strongly agree). Previous studies show that the scale of the planned behaviour theory is valid and reliable and has been used in Estonia [25,26].

#### 2.2.7. Self-Reported Physical Activity

A simple two-question questionnaire (Leisure-Time Exercise Questionnaire [39]) was used to measure subjective physical activity. Participants responded to two items: “How frequently have you participated in vigorous physical activities during your leisure-time in the course of the past five weeks for at least 20 min at a time?”, with responses reported on a six-point scale (one = never and six = all of the time), and “In the course of the past five weeks, how often on average, have you participated in vigorous physical activities during your leisure-time for at least 20 min at a time?” with responses reported on a six-point scale (one = not at all and six = most days per week). Previous research shows that the subjective physical activity questionnaire is valid and reliable and has been used in Estonia [40,41].

#### 2.2.8. Physical Activity Component

A questionnaire with 7 statements was used to measure physical activity [42]. The questionnaire measured students’ activity during the last 7 days. The questionnaire measured the minutes spent on strenuous physical activity (e.g., running, football, basketball), moderate strenuous physical activity (e.g., leisurely skating, rollerblading, Nordic walking), moderate physical activity (walking), and contented activities. The minutes spent on the respective activities were multiplied by the metabolic coefficient of the respective activities. Metabolic Equivalent of Task (MET) refers to the consumption of oxygen over a period of time related to physical activity: 1 MET = 3.5 mL/kg/min. The more intense the activity, the higher the oxygen consumption. The minutes of strenuous physical activity were multiplied by eight, the minutes of moderately strenuous physical activity were multiplied by four, the minutes of moderate physical activity were multiplied by three, and the minutes of relaxation (e.g., sitting) were multiplied by one. The numbers obtained were added together to obtain a component representing the student’s physical activity. A previous study showed that the questionnaire examining the component of physical activity is valid and reliable [25].

### 2.3. Data Analysis

IBM SPSS Statistics 23 and IBM SPSS AMOS 23 were used for statistical analysis of the data. The arithmetic mean and standard deviation were found for all characteristics. The normal distribution of data was checked, with conditions ranging from −10 to +10 for kurtosis and −3 to +3 for skewness when using SEM [43]. The reliability of the scales used was evaluated based on Cronbach’s alpha coefficient, which should be between 0.6 and 0.95 [44]. Pearson correlation analysis was used to assess correlations among study variables. *T*-test for independent variables was used to assess differences between gender groups.

For AMOS, a confirmatory factor analysis was performed for each instrument with the following fit index values within the acceptable range: Comparative fit index (CFI) and Bentler–Bonett non-normal fit index (NNFI) value >0.90 and root mean square error of approximation (RMSEA) value <0.08 [45].

The model used in the study was tested using variation-based structural equation modelling (VB-SEM), also known as Partial Least Squares (PLS) analysis, using Warp PLS v7.0 software [46]. VB-SEM is a distribution-free analytical method used in the past that has shown that model complexity, abnormality, and smaller sample sizes affect research less [47]. The overall suitability of the model in the VB-SEM analysis was assessed based on several criteria: goodness of fit (GoF) values, low ≥ 0.100, mean ≥ 0.250, and high ≥ 0.360 [48]; Average Variance Inflation Factor (AVIF) value, expected to be less than 5000 [46]; Average Path Coefficient (APC); and average R^2^ (ARS).

## 3. Results

### 3.1. Descriptive Statistics on the Characteristics Measured in the Study

The structural model used in the research uses 14 indicators. Table 1 shows the means, standard deviations, reliability values, and normal distribution of the variables of the study based on the values of the skewness and kurtosis values. All study variables have skewness values in the range of −3 to +3 and kurtosis values in the range of −10 to +10, meaning that all traits are within the acceptable range for normal distribution [43]. Cronbach’s alpha is predominantly above 0.70, indicating the reliability of the questionnaires used. Controlled motivation in the physical education questionnaire responses gave a Cronbach’s alpha score of 0.67, but this is in the acceptable range of 0.6 to 0.7 [44].

### 3.2. Results of a Confirmatory Factor Analysis

Table 2 shows the final results of the confirmatory factor analysis for the measuring instruments used in the study. Initially, the results of the factor analysis of all measuring instruments were unsuitable according to the RMSEA suitability index. Covariance was added for each instrument.

The perceived internally and externally controlling teaching scale for the initial RMSEA fitness index was 0.097. A covariation was added for the following characteristics: 1. “My physical education teacher punishes me” and “My physical education teacher shows that I am personally hurt if I do not meet his expectations”; 2. “My physical education teacher counts numbers in descending order (10, 9, 8, … 3, 2, 1) that I will definitely continue”, and “My physical education teacher threatens to stop pleasant activities if I do not cooperate ”; and 3. “My physical education teacher pays less attention to me if I disappoint him” and “My physical education teacher often shows that he is disappointed in me”. The final results for the measure “Perceived internal and external control teaching” are provided in Table 2.

The perceived multidimensional controlling teaching scale for the initial RMSEA fitness index was 0.1. A covariation was added for the following characteristics: 1. “My physical education teacher uses grades to make me work harder” and “My physical education teacher pays less attention to me when I have offended him”; 2. “My physical education teacher uses intimidation to I would do what he wants” and “My physical education teacher is less friendly to me if I don’t work out the way he wants”. The final results of the suitability indices for the measure “Perceived multidimensional controlling teaching” are provided in Table 2.

The baseline RMSEA fit index for the basic psychological need frustration scale was 0.091. A covariance was added for the following characteristics: 1. “In physical education, I felt most of the exercises and tasks I just had to do” and “In physical education, I felt pressured to do too much exercise”; 2. “In physical education, I felt pressured to do too much exercise” and “In physical education, I felt insecure about my abilities”. The final results of the suitability indices for the measure “Basic psychological need frustration” are provided in Table 2.

The original RMSEA fit index of the perceived autonomous and controlled motivation in physical education scale was 0.098. A covariate was added for the following characteristics: 1. “I take part in physical education class so that the teacher does not make fun of me” and 2. “I take part in physical education class because I would feel bad if the teacher thinks that I am not good at physical education”. The final results of the fit indices for the measuring tool “Perceived autonomous and controlled motivation in physical education” are provided in Table 2.

The original fit indices for the perceived autonomous and controlled motivation in leisure-time scale were CFI = 0.885, NNFI = 0.771, and RMSEA = 0.221. A covariate was added for the following characteristics: 1. “I am physically active in my leisure time because I enjoy physical activity” and “I am physically active in my leisure time because it is fun”; 2. “I am physically active in my leisure time because I am known people wouldn’t be happy with me if I wasn’t physically active” and “I’m physically active in my spare time because I feel guilty when I’m not physically active”; 3. “I’m physically active in my spare time because people who know me aren’t happy with me if I’m not physically active” and “I’m physically active in my leisure-time because it’s important to me to be physically active”; 4. “I’m physically active in my leisure-time because people who know me are not happy with me if I’m not physically active” and “I am physically active in my leisure-time because I feel pressured by people who know me to be physically active”. The results of the fit indices for the measuring instrument “Perceived autonomous and controlled motivation in leisure-time” are provided in Table 2.

The final results show that the suitability indices for the measuring instruments are at an acceptable level.

### 3.3. Gender Differences in the Variables Used in the Study

Table 3 presents the differences between girls and boys in the variables used in the study. Table 3 presents the standard deviations, means, and *t*-test values of the independent variables. Among the variables of the study, there were statistically significant differences between boys and girls in externally controlling behaviour, the frustration of basic psychological needs, autonomous motivation in physical education, attitude, and perceived behavioural control.

### 3.4. Correlation Analysis Results for the Variables Used in the Study

Table 4 presents the correlative relations between the study characteristics. The results revealed that the dimensions of controlling behaviour were positively and statistically significantly related (r = 0.55–0.79; *p* < 0.01). Controlling behaviour dimensions were positively and statistically significantly related to basic psychological need frustration (r = 0.46–0.61; *p* < 0.01). Basic psychological need frustration was negatively and statistically significantly related to autonomous motivation in physical education (r = −0.17; *p* < 0.01) and positively and statistically significantly related to controlled motivation in physical education (r = 0.51; *p* < 0.01). Autonomous motivation in physical education was positively and statistically significantly related to autonomous motivation in leisure-time (r = 0.71; *p* < 0.01). Controlled motivation in physical education was positively and statistically significantly related to controlled motivation in leisure-time (r = 0.30; *p* < 0.01). Autonomous and controlled motivation in leisure-time was positively and statistically significantly related to attitude, perceived behavioural control, and subjective norms (r = 0.16–0.64; *p* < 0.01). Attitude, perceived behavioural control, and subjective norms were positively and statistically significantly related to intention (r = 0.38–0.74; *p* < 0.01). Intention and perceived behavioural control were positively and statistically significantly related to leisure-time physical activity (r = 0.26–0.28; *p* < 0.01). Subjective physical activity was negatively and statistically significantly related to basic psychological needs (r = −0.19; *p* < 0.01) and positively and statistically significantly related to autonomous motivation in physical education, autonomous motivation in leisure-time, controlled motivation in leisure-time, attitude, perceived behavioural control, subjective norm, and intention (r = 0.27–0.64; *p* < 0.01).

### 3.5. Variation-Based Structural Model

**Figure 1:**
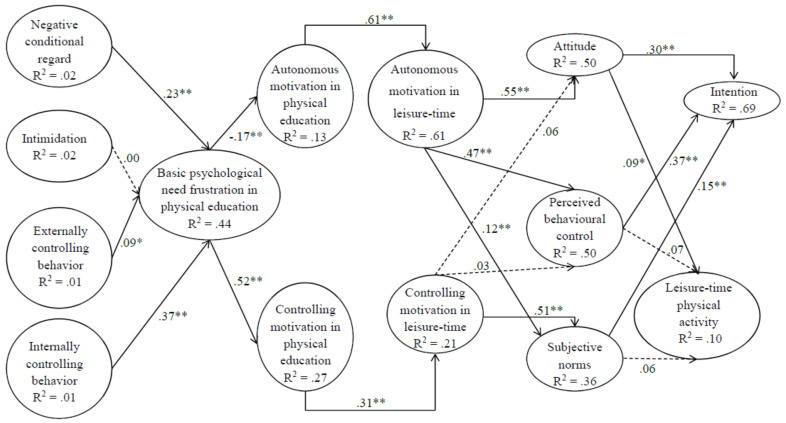
Structural model results. Note: For clarity, the effect of previous physical activity is excluded from the figure, although it is taken into account in the model; statistically significant direct correlations are shown in the figure with a solid line, and statistically non-significant direct correlations are represented with a dotted line; R^2^ = described variance; ** *p* ≤ 0.01.

The results of the variance-based structural model are presented in Figure 1. The fit indices of the variance-based structural model were at a good level: GoF = 0.525, APC =.241, *p* < 0.001, ARS = 0.276, *p* < 0.001, AVIF = 1.589.

The direct relationships of the model are presented in Figure 1. The results revealed that basic psychological need frustration is associated with negative conditional regard (β = 0.23; *p* < 0.01), externally controlling behaviour (β = 0.09; *p* < 0.01), and internally controlling behaviour (β = 0.37; *p* < 0.01). Basic psychological need frustration was statistically significantly related to autonomous motivation in physical education (β = −0.17; *p* < 0.01) and controlled motivation in physical education (β = 0.52; *p* < 0.01). Autonomous motivation in physical education is statistically significantly related to autonomous motivation in leisure-time (β = 0.61; *p* < 0.01), which in turn is statistically significantly related to attitude (β = 0.55; *p* = 0.01), perceived behavioural control (β = 0.47; *p* < 0.01), and subjective norms (β = 0.12; *p* < 0.01). Controlled motivation was statistically significantly related to subjective norm (β = 0.51; *p* < 0.01). Attitude predicted intention (β = 0.30; *p* < 0.01). Perceived behavioural control predicted intention (β = 0.37; *p* < 0.01), and subjective norms predicted intention (β = 0.15; *p* < 0. 01). Attitude was statistically significantly related to the leisure-time physical activity (β = 0.09; *p* < 0.05). The model described 10% of the variability of the leisure-time physical activity representing students’ physical activity.

Indirect relationships are presented in Appendix A. An analysis of the variance-based structural model showed that controlling behaviour was statistically significantly related to controlled motivation in physical education (β = 0.19; *p* < 0.001), which, in turn, was statistically significantly related to subjective norms (β = 0.15; *p* < 0.001). Controlling behaviour was statistically significantly related to controlled motivation in leisure-time (β = 0.06; *p* < 0.05), which, in turn, was statistically significantly related to intention (β = 0.11; *p* < 0.05). Negative conditional regard was negatively and statistically significantly related to controlled motivation in physical education (β = −0.12; *p* < 0.01). Basic psychological need frustration was negatively and statistically significantly related to autonomous motivation in leisure-time (β = −0.11; *p* < 0.01) and statistically significantly related to controlled motivation in leisure-time (β = 0.16; *p* < 0.001). Autonomous motivation in physical education was statistically significantly related to attitude (β = 0.34; *p* < 0.001), perceived behavioural control (β = 0.29; *p* < 0.001), subjective norms (β = 0.08; *p* < 0.05), and intention (β = 0.22; *p* < 0.001). Autonomous motivation in leisure-time was statistically significantly related to intention (β = 0.35; *p* < 0.001).

## 4. Discussion

The aim of this research was to examine how the different dimensions of controlling behaviours predict students’ basic psychological need frustration, motivation, and physical activity in physical education.

The first task in the research was to examine how the different dimensions of the EICT questionnaire predict students’ basic psychological need frustration. In the present work conducted in Estonia, a positive relationship was found between both perceived internally controlling behaviour (β = 0.37) and basic psychological need frustration and between externally controlling behaviour (β = 0.09) and basic psychological need frustration. The results are comparable to the study conducted in the Spanish context by Burgueño and colleagues [28], where perceived internally controlling behaviour predicted psychological need frustration (β = 0.41) and externally controlling behaviour predicted psychological need frustration (β = 0.18). These results indicate that Estonian students’ might not perceive internally and externally controlling behaviours that much, frustrating their psychological needs, as in the Spanish students’ experience. Both results indicate that the coefficient of perceived internally controlling behaviour is slightly stronger than that of perceived externally controlling behaviour. This finding might indicate that internally controlling behaviours are more detrimental to students’ psychological needs because they tend to frustrate more students’ psychological needs. In both studies, the positive relationship between perceived internally and externally controlling behaviour and basic psychological need frustration indicates that teachers’ controlling behaviour can lead to a decrease in autonomous forms of motivation [49]. The effect is larger for perceived internally controlling behaviour, meaning that students perceive internal control more than external control in predicting basic psychological need frustration. Thus, in order to provide autonomous motivation and avoid increasing controlled motivation in students, we recommend avoiding all forms of controlling behaviours, specifically internally controlling behaviours, which can lower student autonomous motivation.

The second task of this research was to examine how the EICT questionnaire and the CCBS questionnaire predict the components of the trans-contextual model of motivation. According to the variance-based structural model, negative conditional regard and internally and externally controlling behaviour are positively related to basic psychological need frustration. Based on this, it is likely that when a PE teacher is less supportive of students when they do not exercise and perform well (i.e., negative conditional regard), count down aloud to make sure students persist (i.e., externally controlling behaviour), and pay less attention to students if they disappoint the teacher (i.e., internally controlling behaviour), then students might experience high levels of basic psychological need frustration. This finding is comparable to a previous study in which it was found that perceived intimidation frustrates students’ psychological needs [17]. The current study adds to our knowledge by additionally examining the effect of internally and externally controlling behaviours on psychological need frustration. This knowledge allows us to create more specific guidelines for PE teachers, such as what kind of behaviours should be avoided. According to the model, there was no statistically significant relationship between intimidation and basic psychological need frustration in this work. One possible reason for this might be that intimating behaviour is usually focused only on one student, not on the whole group. Thus, PE teachers can intimate only one student at once, not the whole group together, which, in turn, means this kind of behaviour occurs more rarely per student. Negative conditional regard (β = 0.23) and perceived internally controlling behaviour (β = 0.37) had stronger effects than externally controlling behaviour (β = 0.09) or intimidation (β = 0.00). This similarity in the strength of effects may be due to the fact that students are more influenced by perceived internal control towards psychological frustration. Negative conditional regard and perceived internally controlling behaviour were also indirectly statistically significantly related to controlled motivation in physical education. This finding is partly similar to previous studies [32] and indicates that controlling behaviours should be avoided if the aim is to avoid creating the experience of controlled motivation in students.

The third task of this research was to examine how the different dimensions of the EICT and CCBS questionnaires predict student motivation in physical education through the frustration of students’ basic psychological needs in physical education. The results of the work showed that basic psychological need frustration was negatively and statistically significantly (β = −0.17) related to autonomous motivation in physical education, and basic psychological need frustration was positively and statistically significantly (β = 0.52) related to controlled motivation in physical education. This finding indicates that when students experience this, their psychological needs are frustrated; thus, students also tend to experience more controlled forms of motivation, a finding similar to previous studies [32]. This is also supported by self-determination theory [3], according to which basic psychological need frustration implies low intrinsic motivation. The positive and statistically significant relationship between basic psychological need frustration and controlled motivation in physical education is similar to another study [31]; basic psychological need frustration was similarly statistically significantly related to controlling teaching, but also in relation to amotivation and controlled motivation. The current study adds our knowledge by investigating the further motivational links and explains the mechanism by which perceived controlling behaviours relate to adolescents’ leisure-time physical activities. Such results confirm that perceived controlling behaviour by students might cause a decrease in students’ autonomous motivation, and, thus, the emphasis should be on supporting students’ autonomy, which, in turn, enhances their intrinsic motivation.

The fourth task of this research was to examine how the different dimensions of the EICT and CCBS questionnaires predict students’ physical activity in their leisure-time through need frustration and the motivation of students’ basic psychological needs in physical education. Basic psychological need frustration was negatively, but not statistically significantly, related to intention and perceived behavioural control. The reason for this might be that there are other factors underpinning this process that are more dominant, such as basic psychological need satisfaction, which was not measured in this study. Beyond basic psychological need frustration, the relationships between physical education motivation and leisure motivation were similar to the study conducted in [26], where autonomous motivation in physical education predicted attitude and perceived behavioural control, and these, in turn, predicted intention. The reason for this might be that autonomously motivated students form their intentions towards physical activity via perceived attitudes and perceived behavioural control. Attitudes and perceived behavioural control also predicted leisure-time physical activity. However, subjective norms did not predict leisure-time physical activity. These findings indicate perceived attitudes and perceived behavioural control are more proximal predictors of leisure-time physical activity, rather than subjective norms. The reason for this might be that adolescents draw decisions related to leisure-time physical activity behaviour based on how enjoyable, good, and useful (i.e., attitudes) they perceive the action to be and how much control they perceive to have to be physically active (i.e., perceived behavioural control). Controlled motivation predicted only subjective norms in both studies. The reason for this might be that the content of subjective norms such as how much adolescents think that the other people who are important to them think they should be physically active is more similar to the content of controlled motivation. From here, it can be concluded that perceived controlled motivation in physical education has a rather minor effect on physical activity in leisure-time. In contrast, autonomous motivation in physical education supports relatively more physically active behaviour in the context of leisure-time.

As a sub-goal of this work, the gender differences in the study variables were examined. Differences were found in externally controlling behaviour, basic psychological need frustration, autonomous motivation in physical education, attitudes, and perceived behavioural control. Girls reported significantly lower externally controlling behaviour and higher psychological need frustration in PE compared to boys. This might indicate that girls are more sensitive to controlling behaviours compared to boys. In addition, girls reported significantly lower autonomous motivation in PE and lower levels of attitudes and perceived behavioural control compared to boys. This might indicate that if girls’ psychological needs are relatively more frustrated, then their autonomous motivation is also relatively lower compared to boys. This, in turn, might result in lower levels of attitudes and perceived behavioural control. Girls also reported a trend regarding lower levels of leisure-time physical activity compared to boys; however, these differences remained insignificant. Several limitations had to be taken into account based on the results of this research. First, the number of participants included in the study was relatively small (*n* = 299). Since a large number of different indicators were evaluated in the model, the number of participants could have been bigger. Another limitation was the subjectivity of the self-reported survey. Respondents could understand the questions differently and give unthought-out answers. Moreover, it might be difficult for students to recall how long they take in MVPA. Future studies could adopt more objective measurement methods for data collection. In addition, a large amount of variance remained unexplained in the current study. Future studies could adopt other prominent indicators that are related to leisure-time physical activity, such as perseverance effort and consistent interest [40].

### Practical Applications

In summary, based on the results of the current study, several practical recommendations could be drawn. PE teachers are highly recommended to avoid controlling behaviours, such as being less friendly with students if he or she does not make as much effort as the teacher wants (i.e., negative conditional regard), threatening to punish students if they do not practise in class (i.e., intimidation), counting down aloud to make sure students persist (i.e., externally controlling behaviour), and paying less attention to students if they disappoint the teacher (i.e., internally controlling behaviour). The reason to avoid such behaviours is that students might experience basic psychological need frustration and, in turn, experience higher levels of controlled motivation. When students experience controlled motivation, then it is more likely that students will be less physically active. It is also important to note that girls tend to experience higher levels of basic psychological need frustration. This might indicate that girls are more sensitive to controlling behaviours.

## 5. Conclusions

Based on the results of this research, the following conclusions were drawn:

1. The perceived internal control component of the EICT questionnaire has a stronger relationship with basic psychological need frustration than the perceived external control component.
2. The perceived internal control component of both the EICT questionnaire and the CCBS questionnaire shows a stronger relationship with basic psychological need frustration than the component of external behaviour. In other words, students perceive more internal behaviour than external behaviour when basic psychological needs are frustrated. The components characterizing internal control in both questionnaires were indirectly and strongly related to controlling motivation in physical education.
3. Students’ basic psychological need frustration can promote more controlled motivation in physical education and can reduce autonomous motivation in physical education.
4. The more the controlling teaching is perceived by the students, the lower the levels of physical activity are in the students in the context of leisure-time.
5. Girls perceive the frustration of basic psychological needs higher than boys.

## Figures and Tables

**Table 1:** Descriptive statistics on survey characteristics (*n* = 299).

Characteristic	M ± SD	Skewness	Kurtosis	Cronbach α
Internally controlling behaviour	1.60 ± 1.05	1.64	2.55	0.91
Externally controlling behaviour	2.05 ± 1.13	2.00	4.45	0.80
Negative conditional regard	1.74 ± 1.26	2.15	3.36	0.87
Intimidation	1.45 ± 1.02	3.02	9.53	0.89
Basic psychological need frustration in PE	2.47 ± 1.23	1.02	0.75	0.91
Autonomous motivation in PE	5.06 ± 1.70	−0.70	−0.42	0.90
Controlling motivation in PE	3.10 ± 1.44	0.40	−0.53	0.67
Autonomous motivation in leisure-time	5.01 ± 1.80	−0.68	−0.54	0.94
Controlling motivation in leisure-time	3.37 ± 1.55	0.34	−0.51	0.78
Attitude	5.24 ± 1.52	−0.63	−0.07	0.91
Perceived behavioural control	4.86 ± 1.65	−0.45	−0.55	0.86
Subjective norm	3.55 ± 1.69	0.29	−0.69	0.78
Intention	4.80 ± 1.80	−0.29	−0.97	0.94
Subjective physical activity	3.64 ± 1.28	−0.05	−0.42	0.87

Note: M—mean; SD—standard deviation; α—Cronbach’s α; PE—physical education.

**Table 2:** Results of a confirmatory factor analysis.

Study Constructs	*χ* ^2^	*df*	CFI	NNFI	RMSEA
Perceived internally and externally controlling teaching	140.731	50	0.955	0.940	0.078
Perceived multidimensional controlling teaching	61.402	22	0.977	0.963	0.078
Basic psychological need frustration	139.057	49	0.961	0.947	0.079
Perceived autonomous and controlled motivation in physical education	32.077	13	0.984	0.966	0.070
Perceived autonomous and controlled motivation in leisure-time	218.218	14	0.995	0.987	0.053
Theory of planned behaviour	46.569	19	0.987	0.975	0.070

Note: *χ*^2^—chi-square; *df*—degrees of freedom; CFI—comparative fit index; NNFI—Bentler–Bonnet non-normed fit index; RMSEA—root-mean-square error of approximation.

**Table 3:** Gender differences in study variables.

Study Variables	Boys (*n* = 164)	Girls (*n* = 135)	
	M ± SD	M ± SD	t-Value
Internally controlling behaviour	1.58 ± 0.95	1.62 ± 1.17	−0.32
Externally controlling behaviour	2.28 ± 1.24	1.77 ± 0.92	3.99 *
Negative conditional regard	1.73 ± 1.21	1.76 ± 1.33	−0.20
Intimidation	1.48 ± 0.98	1.41 ± 1.08	0.60
Basic psychological need frustration in PE	2.29 ± 1.10	2.69 ± 1.35	−2.79 *
Autonomous motivation in PE	5.26 ± 1.69	4.82 ± 1.68	2.25 *
Controlling motivation in PE	3.00 ± 1.45	3.23 ± 1.43	−1.38
Autonomous motivation in leisure-time	5.16 ± 1.76	4.84 ± 1.85	1.51
Controlling motivation in leisure-time	3.38 ± 1.55	3.35 ± 1.56	0.15
Attitude	5.45 ± 1.47	4.97 ± 1.54	2.75 *
Perceived behavioural control	5.03 ± 1.69	4.66 ± 1.57	1.97 *
Subjective norm	3.71 ± 1.61	3.35 ± 1.76	1.86
Intention	4.96 ± 1.82	4.61 ± 1.77	1.65
Subjective physical activity	3.70 ± 1.29	3.58 ± 1.26	0.74
Leisure-time physical activity	4056.35 ± 4389.52	3433.79 ± 2953.55	1.41

Note: M—mean; SD—standard deviation; PE—physical education; t-value—*t*-test value of independent variables; *—*p* < 0.05.

**Table 4:** Relationships between study variables.

	Correlation														
	Study Variables	1.	2.	3.	4.	5.	6.	7.	8.	9.	10.	11.	12.	13.	14.
1.	Internally controlling behaviour	1													
2.	Externally controlling behaviour	0.62 **	1												
3.	Negative conditional regard	0.79 **	0.55 **	1											
4.	Intimidation	0.75 **	0.65 **	0.74 **	1										
5.	Basic psychological need frustration in PE	0.61 **	0.46 **	0.57 **	0.51 **	1									
6.	Autonomous motivation in PE	−0.13 *	0.00	−0.10	−0.14 *	−0.17 **	1								
7.	Controlling motivation in PE	0.42 **	0.35 **	0.41 **	0.34 **	0.51 **	0.14 *	1							
8.	Autonomous motivation in leisure-time	−0.07	−0.01	−0.07	−0.10	−0.17 **	0.71 **	0.06	1						
9.	Controlling motivation in leisure-time	0.16 **	0.13 *	0.17 **	0.12 *	0.19 **	0.29 **	0.30 **	0.48 **	1					
10.	Attitude	−0.19 **	−0.07	−0.15 *	−0.20 **	−0.27 **	0.56 **	−0.07	0.64 **	0.29 **	1				
11.	Perceived behavioural control	−0.00	−0.02	−0.03	−0.04	−0.24 **	0.43 **	−0.03	0.62 **	0.31 **	0.65 **	1			
12.	Subjective norm	0.18 **	0.09	0.23 **	0.21 **	0.13 *	0.03	0.21 **	0.16 **	0.51 **	0.17 **	0.28 **	1		
13.	Intention	−0.02	−0.04	−0.01	−0.04	−0.19 **	0.41 **	0.00	0.65 **	0.41 **	0.68 **	0.74 **	0.38 **	1	
14.	Subjective physical activity	0.03	0.00	0.04	0.04	−0.19 **	0.27 **	−0.02	0.49 **	0.34 **	0.50 **	0.56 **	0.30 **	0.64 **	1
15.	Leisure-time physical activity	0.05	0.09	0.04	0.01	0.00	0.11	0.03	0.19 **	0.14 *	0.22 **	0.21 **	0.16 **	0.26 **	0.28 **

Note: PE—physical education, * *p* < 0.05, ** *p* < 0.01.

## Data Availability

Dataset can be found here https://osf.io/vnzjc/?view_only=4bac4cce1eec41aea825e050e28792bb.

## Appendix A

**Table A1:** Standardized indirect relationships between study variables.

Independent Variable	Dependent Variable	β	*p*-Value
Internally controlling behaviour	Autonomous motivation in physical education	−0.063	0.06
Internally controlling behaviour	Controlled motivation in physical education	0.189	<0.001
Internally controlling behaviour	Autonomous motivation in free time	−0.039	0.122
Internally controlling behaviour	Controlled motivation in free time	0.058	0.040
Internally controlling behaviour	Attitude	−0.018	0.334
Internally controlling behaviour	Perceived behavioural control	−0.016	0.344
Internally controlling behaviour	Subjective norm	0.025	0.272
Internally controlling behaviour	Intention	−0.007	0.449
Internally controlling behaviour	Leisure-time physical activity	−0.001	0.488
Externally controlling behaviour	Autonomous motivation in physical education	−0.016	0.346
Externally controlling behaviour	Controlled motivation in physical education	0.048	0.118
Externally controlling behaviour	Autonomous motivation in free time	−0.010	0.383
Externally controlling behaviour	Controlled motivation in free time	0.015	0.328
Externally controlling behaviour	Attitude	−0.018	0.334
Externally controlling behaviour	Perceived behavioural control	−0.016	0.344
Externally controlling behaviour	Subjective norm	0.025	0.272
Externally controlling behaviour	Intention	−0.007	0.449
Externally controlling behaviour	Leisure-time physical activity	−0.001	0.488
Intimidation	Autonomous motivation in physical education	0.000	0.499
Intimidation	Controlled motivation in physical education	−0.000	0.496
Intimidation	Autonomous motivation in free time	0.000	0.499
Intimidation	Controlled motivation in free time	−0.000	0.498
Intimidation	Attitude	0.000	0.500
Intimidation	Perceived behavioural control	0.000	0.500
Intimidation	Subjective norm	−0.000	0.499
Intimidation	Intention	0.000	0.500
Intimidation	Leisure-time physical activity	0.000	0.500
Negative Conditional regard	Autonomous motivation in physical education	−0.040	0.163
Negative Conditional regard	Controlled motivation in physical education	0.119	0.002
Negative Conditional regard	Autonomous motivation in free time	−0.025	0.231
Negative Conditional regard	Controlled motivation in free time	0.037	0.134
Negative Conditional regard	Attitude	−0.011	0.393
Negative Conditional regard	Perceived behavioural control	−0.010	0.400
Negative Conditional regard	Subjective norm	0.016	0.351
Negative Conditional regard	Intention	−0.005	0.468
Negative Conditional regard	Leisure-time physical activity	−0.001	0.493
Basic psychological need frustration	Autonomous motivation in free time	−0.106	0.004
Basic psychological need frustration	Controlled motivation in free time	0.159	<0.001
Basic psychological need frustration	Attitude	−0.048	0.154
Basic psychological need frustration	Perceived behavioural control	−0.045	0.171
Basic psychological need frustration	Subjective norm	0.068	0.075
Basic psychological need frustration	Intention	−0.020	0.364
Basic psychological need frustration	Leisure-time physical activity	−0.003	0.472
Autonomous motivation in physical education	Attitude	0.336	<0.001
Autonomous motivation in physical education	Perceived behavioural control	0.288	<0.001
Autonomous motivation in physical education	Subjective norm	0.076	0.031
Autonomous motivation in physical education	Intention	0.217	<0.001
Autonomous motivation in physical education	Leisure-time physical activity	0.019	0.287
Controlled motivation in physical education	Attitude	0.020	0.314
Controlled motivation in physical education	Perceived behavioural control	0.010	0.403
Controlled motivation in physical education	Subjective norm	0.156	<0.001
Controlled motivation in physical education	Intention	0.034	0.280
Controlled motivation in physical education	Leisure-time physical activity	0.001	0.492
Autonomous motivation in leisure-time	Intention	0.353	<0.001
Autonomous motivation in leisure-time	Leisure-time physical activity	0.031	0.227
Controlled motivation in leisure-time	Intention	0.109	0.028
Controlled motivation in leisure-time	Leisure-time physical activity	0.002	0.479
